# Obesity impairs resistance to *Leishmania major* infection in C57BL/6 mice

**DOI:** 10.1371/journal.pntd.0006596

**Published:** 2020-01-10

**Authors:** Vinicius Dantas Martins, Franciele Carolina Silva, Felipe Caixeta, Matheus Batista Carneiro, Graziele Ribeiro Goes, Lícia Torres, Sara Cândida Barbosa, Leonardo Vaz, Nivea Carolina Paiva, Cláudia Martins Carneiro, Leda Quercia Vieira, Ana Maria Caetano Faria, Tatiani Uceli Maioli

**Affiliations:** 1 Programa de Pós-Graduação em Bioquímica e Imunologia, Instituto de Ciências Biológicas, Universidade Federal de Minas Gerais, Belo Horizonte, Brazil; 2 Programa de Pós-Graduação Interunidades de Bioinformática, Universidade Federal de Minas Gerais, Belo Horizonte, Brazil; 3 Snyder Institute for Chronic Diseases, Departments of Microbiology, Immunology and Infectious Disease, Cumming School of Medicine, University of Calgary, Alberta, Canada; 4 Núcleo de Pesquisa em Ciências Biológicas, Instituto de Ciências Exatas e Biológicas, Universidade Federal de Ouro Preto, Ouro Preto, Brazil; 5 Departamento de Nutrição, Escola de Enfermagem, Universidade Federal de Minas Gerais, Belo Horizonte, Brazil; McGill university, CANADA

## Abstract

An association between increased susceptibility to infectious diseases and obesity has been described as a result of impaired immunity in obese individuals. It is not clear whether a similar linkage can be drawn between obesity and parasitic diseases. To evaluate the effect of obesity in the immune response to cutaneous *Leishmania major* infection, we studied the ability of C57BL/6 mice fed a hypercaloric diet (HSB) to control leishmaniasis. Mice with diet-induced obesity presented thicker lesions with higher parasite burden and a more intense inflammatory infiltrate in the infected ear after infection with *L*. *major*. There was no difference between control and obese mice in IFN-gamma or IL-4 production by auricular draining lymph node cells, but obese mice produced higher levels of IgG1 and IL-17. Peritoneal macrophages from obese mice were less efficient to kill *L*. *major* when infected *in vitro* than macrophages from control mice. *In vitro* stimulation of macrophages with IL-17 decreased their capacity to kill the parasite. Moreover, macrophages from obese mice presented higher arginase activity. To confirm the role of IL-17 in the context of obesity and infection, we studied lesion development in obese IL-17R-/- mice infected with *L*. *major* and found no difference in skin lesions and the leukocyte accumulation in the draining lymph node is redcuced in knockout mice compared between obese and lean animals. Our results indicate that diet-induced obesity impairs resistance to *L*. *major* in C57BL/6 mice and that IL-17 is involved in lesion development.

## Author summary

Obesity is a serious and increasing public health problem, and also induces a spectrum of metabolic disorders. Some diseases are known to be more severe in the presence of obesity. However, the interactions of obesity with the immune response to infectious agents have not been fully explored. In this study, we investigated the response of obese mice to infection with *Leishmania major*. C57BL/6 mice were fed a hypercaloric diet (HSB) and infected afterward with *L*. *major*. In obese mice, lesions were ticker and more ulcerative, and cells from draining lymph nodes produced more IL-17 when compared with cells from lean mice fed a control diet. Macrophages from obese and lean mice were infected *in vitro* and stimulated with IL-17 to test the role of this cytokine in effect produced by obesity. Macrophages from obese mice were more infected by *L*. *major* than the macrophages from control mice and the number of parasites was increased by treatment with IL-17. IL-17R deficient mice treated with hypercaloric diet showed no difference in lesion size when compared to mice fed control diet. Our findings suggest that diet-induced obesity decrease the resistance to *L*. *major* infection of C57BL/6 mice and the IL-17 cytokine may be involved in the lesion formation.

## Introduction

Obesity is characterized by excessive fat accumulation, and it is considered a multifactorial chronic disease that has increased over the last decades. It is associated with a metabolic syndrome that includes insulin resistance, type 2 diabetes mellitus, dyslipidemia, and hypertension, and also leads to respiratory diseases, hepatic steatosis, polycystic ovary syndrome, infertility, cancer, stroke, osteoarthritis [1].

Metabolic syndrome and co-morbidities associated with obesity occur in an environment characterized by the presence of chronic low-grade inflammation [2,3]. The link between obesity and inflammation started in the early 1990s when researchers demonstrated that TNF-alfa expression was elevated in adipose tissue from obese individuals and it was related to insulin resistance [4]. Since then, many studies have correlated obesity with inflammation, characterized by macrophage accumulation in adipose tissue [5,6] decrease in dendritic cell (DC) and natural killer cell (NK) functions [7,8]. In addition, obesity alters the profile of T cells in adipose tissue [9]. While T helper 1 (Th1) and CD8+ T-cells are increased in the adipose tissue of obese animals [6], regulatory T cells are reduced [10,11]. These alterations in immune cells may cause alterations in immune responses in obese individuals. However, it is unclear whether inflammation associated with obesity impacts in the immune response to infectious diseases.

It has been shown that obesity increases the susceptibility to infection by different agents such as influenza virus (H1N1) [12], *Helicobacter pylori* [13] and *Staphylococcus aureus* [14]. Karlsson and coworkers showed that obese mice infected with the H1N1 virus had increased inflammation, characterized by higher production of TNF-alfa and IL-6. Nevertheless, these animals had a poor memory CD8+ T-cell response and were more susceptible to infection [15]. Overweight and obese individuals also had a defective immune response to H1N1 viral infection [16].

Other studies have addressed the effect of obesity on parasite infections. There is a positive correlation between obesity and increased incidence of *Toxoplasma gondii* infection [17]. Interestingly, diet-induced obesity in C57BL/6 mice was protective in a model of cerebral malaria [18]. Hypothalamic obesity in C57BL/6 mice infected with *Plasmodium berghei* ANKA resulted in decreased parasitemia, but exacerbated inflammation, and increased mortality rate[19]. Leptin-deficient obese mice (ob/ob mice) are also more susceptible to *Trypanosoma cruzi* infection [20,21]. Sarnáglia showed that diet-induced obesity promoted susceptibility to visceral leishmaniasis followed by higher production of pro-inflammatory cytokines and increased parasite load [22].

In spite of these studies on obesity and parasite infection, the interactions of obesity with leishmaniasis are still poorly understood. Resistance to *Leishmania major* infection is well characterized in C57BL/6 mice. Induction of an early Th1 response is necessary to induce resistance. Initial activation of dendritic cells leads to the production of IL-12 [23] that promotes a Th1 response with high levels of IFN-gamma and TNF-alfa production, and low levels of IgG1 antibody secretion. The establishment of this polarized inflammatory environment activates the expression of iNOS and NO production in macrophages, which has leishmanicidal activity [24,25]. This is a well-established model of resistance to infection. Therefore, we decided to investigate if obesity would interfere in the outcome of cutaneous leishmaniasis in C57BL/6 mice.

Our results showed that obesity did not affect the development of a Th1 response, nor trigger a Th2 response. However, obese mice were more susceptible to infection. Moreover, the increased IL-17 production found in obese mice in response to infection was not able to control *Leishmania* growth, suggesting that this cytokine could be one reason for parasite growth. Our findings with mice that lack the receptor for IL-17 (IL-17R-/-) indicate that this interleukin is important for the inflammation and control of *L*. *major* infection C57BL/6. The increased inflammation with higher IL-17 production provides one mechanism for the increased susceptibility of obese mice to *L*. *major* infection. Our results bring new insights into the interactions between immune response and obesity in animals facing a parasite infection with possible implications for the clinical outcome of leishmaniasis during obesity.

## Methods

### Animals and diet-induced obesity

All experiments were performed using six-eight-week-old female C57BL/6 mice, weighting approximately 18g, and obtained from the Animal Facility at the Universidade Federal de Minas Gerais (CEBIO, UFMG—Belo Horizonte, Brazil). IL-17 receptor-deficient mice C57BL/6 background (IL-17R-/-) were generated according to http://transgenose.cnrs-orleans.fr/eng/taam/lignees/caracLignees.php?l=1346#top and purchased from Centro de Criação de Camundongos Especiais Faculdade de Medicina de Ribeirão Preto (USP-Ribeirão Preto). All animals were maintained in the Experimental Animal Facility of the Laboratório de Imunobiologia in collective cages (5 animals/cage) in an environmentally controlled room with a 12-hour light/dark cycle, controlled temperature (28°C) and unlimited access to water and food. The obesity was induced with high sugar and butter diet (HSB, calories are 36% from carbohydrates and 48% from lipids), given *ad libitun* to the mice [11]. Other components of the diet were identical to avoid any nutritional deficit in the HSB mice. HSB diet and the control diet (AIN93G) were prepared as described by Reeves, 1993 [26].

### Ethics statement

Procedures and manipulation of animals followed the guidelines of the ethics committees in research of Universidade Federal de Minas Gerais in agreement with guidelines of the ethics committees in research from Brazilian Federal Law #11794, October 8th, 2008: http://www.planalto.gov.br/ccivil_03/_ato2007-2010/2008/lei/l11794.htm. All animal protocols were approved by the Committee on Animal Experiments (CETEA) under the protocol 338/2012, and it was approved in 01/10/2013.

### Experimental design

**Fig 1:**
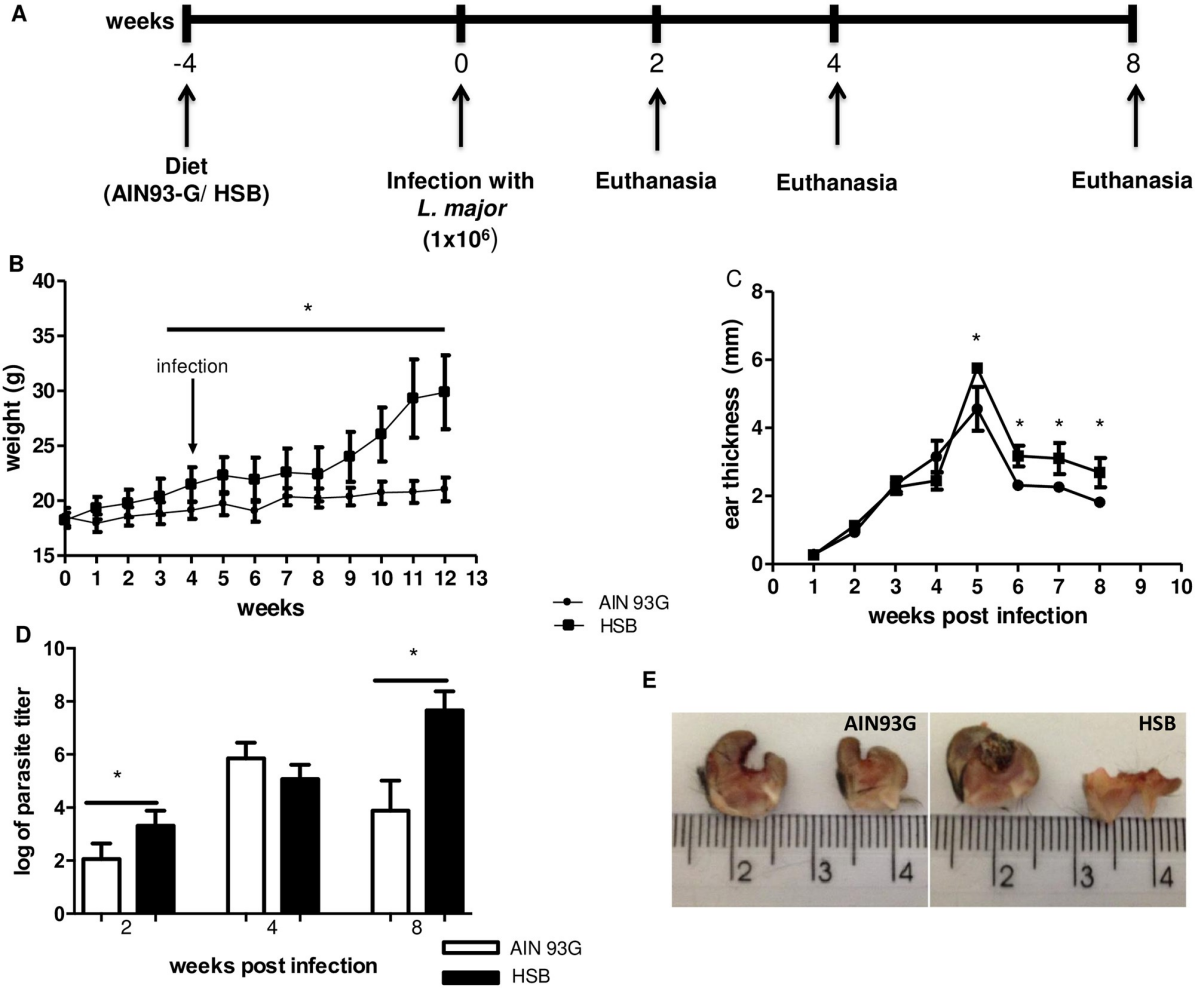
Experimental design and course of infection in diet-induced obese C57BL/6 mice. Animals were fed control (AIN93-G) or hypercaloric (HSB) diets *ad libitum* for four weeks. On the fourth week mice were infected with 1x10^6^ metacyclic *L*. *major* in the ear. Weight gain and food intake were measured weekly. (A) Experimental design. (B) Weekly measurement of C57BL/6 body weight. (C) Weekly measurement of infected ear thickness during eight weeks of infection. (D) Parasite titer in the infected ear, measured by limiting dilution. Data are represented as average ± SD. Statistical analysis was performed by Student´s *t* test (*p<0.05; **p<0.005). Results are representative of at least 4 independent experiments, n = 4 mice/group.

Mice were randomly divided into two groups: control (fed AIN-93G) and obese (fed HSB). Animals were fed the same diet throughout the experiment. On the 4^th^ week of diet consumption, mice were infected with 1x10^6^ metacyclic promastigotes of *Leishmania major*. Infection was followed for 8 weeks. Body weight, lesion size, fasting glycemia, LDL cholesterol and triglycerides levels were measured weekly during this period. Animals were euthanized on the second, fourth and eighth weeks post-infection (Fig 1A).

### Parasites, infection, and antigens

*Leishmania (Leishmania) major* (WHO MHOM/IL/80/Friedlin) were maintained in Grace`s medium (GIBCOBRL—Life Technologies, Grand Island, NY, MO, EUA), pH 6.2 supplemented with 20% of fetal bovine serum (GIBCO), 20 μg/mL gentamicin sulfate (Schering-Plough—Rio de Janeiro, RJ, Brazil) and 2mMl-glutamine (GIBCOBRL—Life Technologies, Grand Island, NY, MO, EUA) (supplemented Grace`s), at 25°C. The metacyclic promastigote forms were separated by Ficoll gradient (Ficoll 400, Sigma-Aldrich, INC., St Louis, MO, USA) at day 5 of culture. In a 15mL conical tube, we added 2mL of Ficoll 20%, then 2mL of Ficoll 10%, without homogenizing the two solutions, forming two distinct phases (Ficoll gradient). Finally, the Leishmania suspension was added by carefully flowing through the wall of the tube forming the third phase. This three-phase mixture was centrifuged at 800 x *g* for 10 min at 4°C. The ring formed and all the supernatant above it, corresponding to the 10% Ficoll and Leishmania phases in PBS, were collected with a Pasteur pipette. In another tube, 10 mL of PBS was added to the supernatant and the suspension was centrifuged at 1500 x *g* for 15 minutes at 4°C. The supernatant was discarded, and the pellet was recovered in 2 mL of PBS. A 1:100 diluted aliquot was withdrawn in PBS containing 4% formalin and the number of parasites was counted in Neubauer's chamber. Parasites (1x10^6^) were inoculated intradermally in the left ear of each animal (final volume = 10μl). Lesion development was monitored weekly by the difference in thickness between infected and uninfected ears, as measured by a digital caliper (Starrett 727, Starrett, Itu, Brazil).

The number of parasites was estimated by limiting dilution as described previously [27]. Briefly, mice were euthanized and the whole ear was removed and cleaned in 70% alcohol. Ears were fragmented with scissors and grinded in a glass tissue grinder. Tissue debris were removed by centrifugation at 50 *g* for 1 min and the supernatant was transferred to another tube and centrifuged at 1,540 *g* for 15 min. Pellet was resuspended in 0.5 mL supplemented Grace’s medium. The parasite suspension was then serially diluted in 10-fold dilutions in duplicates to a final volume of 200 μl in 96-well plates. Pipette tips were replaced for each dilution. Plates were incubated for 10 days at 25°C and examined under an inverted microscope. Results were expressed as the log_10_ of the last dilution in which parasites were detected in 96 wells plates.

*Leishmania* antigen was obtained from logarithmic phase cultures of *L*. *major* promastigotes. Promastigotes were washed twice in PBS and pellets were submitted to seven cycles of freezing in liquid nitrogen followed by thawing at 37°C. The preparations were visually inspected for the presence of intact parasites. The protein content of preparations was assayed by the Lowry method [28] and adjusted to 1 mg/mL protein. Antigen preparation was aliquoted and stored frozen at -20 °C.

### Histology

Ear samples from the injected sites were collected and fixed in 80% methanol and 20% dimethyl sulfoxide (DMSO; Merck, Darmstadt, Germany), embedded in paraffin, cut into 3–5-μm sections and stained with hematoxylin-eosin (H&E-staining) for microscopic analysis. Images were taken with an optical microscope and are shown at 100x magnification.

### Glycaemia and LDL measurement

To access glycemia, animals fasted for 6 hours and blood was collected from the tail vein. Blood glucose was measured with a glucometer and strips (Accu—Chek Performa). To perform the oral glucose tolerance test (OGTT), glucose was given by gavage (2 g/kg). Measurements were performed with a glucometer and strips (Accu—Chek Performa) before glucose gavage and at 15, 30, 60 and 90 minutes later. For fasting glucose and LDL cholesterol measurements, animals fasted for 6 hours and blood was collected. The glycemia and LDL cholesterol levels were measured by enzymatic kit according to the manufacturer’s protocol (Bioclin, Quiabasa, MG, Brazil).

### Lymph node cell isolation and adipose tissue processes

Single-cell suspensions were prepared from draining auricular lymph nodes of the infected ear harvested at 2, 4 or 8 weeks after infection. Cells were placed in tissue culture plates and adjusted to a concentration of 5x10^6^ cells/mL in RPMI Medium 1640 (GIBCO BRL, Grand Island, N.Y., USA) containing 10% of FCS, 2 mM L-glutamine, 50 μM 2-mercapto-ethanol, 100 U/mL penicillin, 100 μg/ mL fungizone, 1 mM sodium pyruvate, 0.1 mM essential amino acids and 25 mM Hepes. They were stimulated with 50 μg/mL *L*. *major* antigen. After 72 h, supernatants were harvested and IFN-gamma, IL-4, IL-10, and IL-17 were measured by ELISA as described. To measure cytokines from adipose tissue, extracts were prepared after collected the peritoneal adipose tissue, then they were washed with PBS and weighed, for each 100 mg of tissue, 1 mL of cold phosphate buffer containing 0.5% BSA and protease inhibitors were added. Extracts were obtained by homogenizing tissues with an electrical tissue homogenizer. Tissue samples were then centrifuged at 3500 *g* for 15 min, and supernatants were collected and stored at -20°C until use. Cytokines were measured as described elsewhere.

### Enzyme-linked immunosorbent assay (ELISA) for cytokines and antibodies

Cytokines were measured in the lymph node cells supernatants and also in adipose tissue extracts. Leptin was measured in the sera. For measurement of cytokines and leptin, plates were coated (50μL/well) with monoclonal antibodies solution against IFN-gamma (Cat#: 900-TM98), TNF-alfa (Cat#: 900-M54), IL -4 (Cat#: 900-M49), IL -10 (Cat#: 900-K53) and IL -17 (Cat#: 900-K392) (PeproTech, NJ, US) and leptin (Cat#: DY498) (R&D Systems MN, US), diluted in PBS and kept overnight, at room temperature. The enzymatic reaction was revealed by incubating the plates with a solution containing 0.2 μL/mL of H_2_O_2_ and 0.5 mg/mL ABTS ((C_18_H_16_N_4_O_6_S_4_-(NH_4_)_2_) (Sigma-Merck, Germany) in 0.1 M citrate buffer pH 5.0 for the development of a dark green color. After this stage, the reactions were stopped by addition of 20μL/well of a solution of SDS 1%. All protocols are developed according to manufactures protocol. The absorbance (λ405 nm) of each well was obtained by an automatic ELISA reader (Molecular Devices Spectra MAX340).

### Anti-leishmania antibodies ELISA

After mice have received anesthesia, blood was collect and sera were separated by blood centrifugation, and the levels of specific antibodies were detected by capture ELISA using plates coated with 200 μg/mL total *L*. *major* antigen (MaxSorp; NUNC, Rochester, NY, USA). For immunoglobulin detection, specific anti-mouse biotinylated antibodies against mouse IgG, IgG1, IgM and IgG2a were used (PharMingen). For IgG sera were diluted 1:100, for all others sera were diluted at 1:10.

### Macrophage culture

Thioglycolate (2%) solution was injected in the animal’s peritoneum, after 8 weeks on diet, to induce macrophage recruitment. Peritoneal exudate cells were obtained after 72 hours. The collected macrophages were incubated in a 24 well plate onto glass coverslips at the concentration of 1x10^5^ cells/mL. Cells were incubated for 2 hours for adhesion. Then they receive the stimulus to prime the macrophages before infection. Cells were stimulated with 1 ng/mL of IFN-gamma, 1 ng/mL of IL-4, 1 ng/mL of IL-17, or 100 ng/mL of LPS. One hour after they received 5:1 total *L*. *major* in the stationary phase per macrophage, without opsonization. *In vitro* infection was analyzed by optical microscopy 4, and 72 hours post infection after instant glass coverslip staining (Panótico, Laborclin, PA, Brazil). The following parameters were analyzed: percentage of infected cells; amastigotes inside infected cells and infection index (percentage of infected macrophages divided by total amastigotes inside cells). For each mice 300 macrophages were counted, then the percentage of infected macrophages and also the number of amastigotes inside the macrophage. For nitric oxide (NO) and arginase activity, cells were incubated in 96 well plates (1x10^6^ cells/mL) and infected with 5:1 *L*. *major*. The supernatant was collected for NO measurement at 72h post-infection, as well as arginase activity.

### Nitric oxide (NO) assay

Supernatants of macrophage cultures were collected 72h post *in vitro* infection. Nitric oxide production was measured as nitrite in culture supernatants using the Griess’ reaction [29].

### Arginase activity

Arginase activity in homogenates of infected macrophages was assayed as described previously [30] with few modifications. About 35μL macrophage homogenate was incubated in 24-well plates with 50μL Triton X-100 and plates were shaken for 30min. Arginase was activated with 50 μL MnCl_2_ (10 mM) and 50 μL of TRIS-HCl (50 mM, pH 7.5) at 55°C for 10 min. Then, 50μL samples were transferred to a fresh 24-well plate containing 25 μL of l-arginine (0.5 mM, pH 9.7) and incubated for 60 min at 37°C. The reaction was stopped by the addition of 400μL of a mixture of acids and water H_2_SO_4_:H_3_PO_4_:H_2_O (1:3:7). Subsequently, 25 μL of 9% 1-phenyl-1,2-propanodione-2-oxime in ethanol was added and the plates were incubated at 95°C for 45 min for color development. Reaction mixtures were read at 540 nm in a spectrophotometer (Molecular Devices). One unit of enzyme activity is defined as the amount of enzyme that catalyzes the formation of 1 μmol of urea/min. A standard curve was performed using urea and the detection limit for the assay was 270 μM of urea.

### Flow cytometry analysis of leukocytes in auricular draining lymph node

Auricular draining lymph nodes were collected, and the cells were homogenized and stained with anti-CD45-APC-cy7 or their isotype control antibodies (BD Pharmingen) diluted in PBS containing 1% BSA. Cells were fixed in 4% formaldehyde, and at least 50,000 events were acquired using a FACSCan (Becton & Dickinson) equipment, and data were analyzed by FlowJo (Treestar, Ashland, OR, USA) and converted to absolute number according the counting of total alive cells.

### Statistical analysis of the data

Data were initially analyzed using the Kolmogorov-Smirnov test to verify normal distribution. Since all data were normally distributed, the Student's *t*-test was used to compare groups. For the statistical analysis in the IL-17R-/- mice we performed one-way ANOVA. The significance level of p<0.05 was adopted.

## Results

### Obesity was associated with larger lesion and higher parasite burden

C57BL/6 mice showed significant weight gain by the 4^th^ experimental week. Obese mice presented a significant increase in body weight when compared to the control group (Fig 1B). The weight gain persisted until the end of the study whereas the control mice reach a plateau at the 6^th^ week. We also confirmed the presence of metabolic syndrome in HSB-fed mice by their high levels of blood glucose, altered glucose tolerance test, high serum levels of LDL cholesterol, triglycerides, and leptin (S1 Fig).

**Fig 2:**
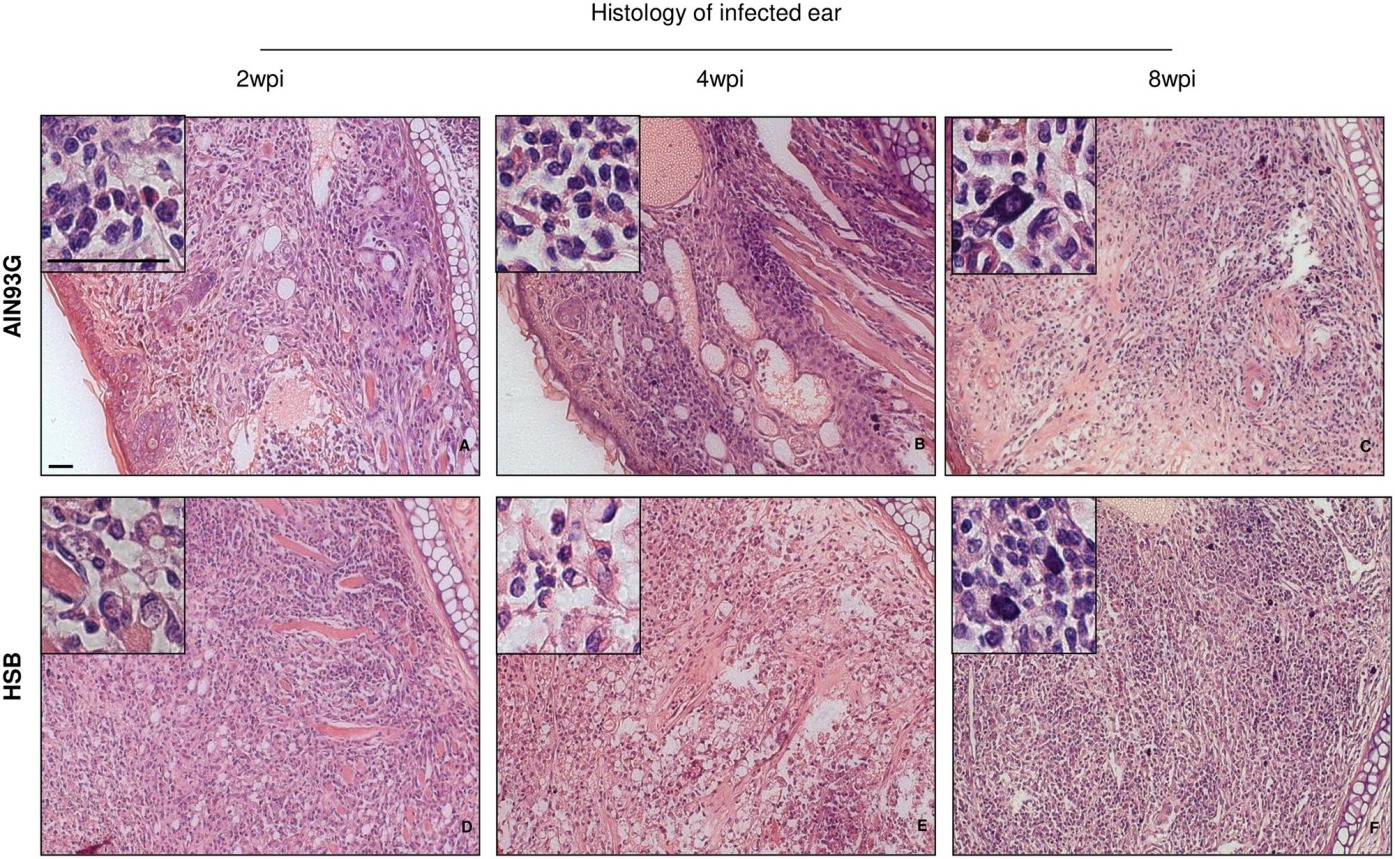
HSB diet-induced obesity leads to a more severe inflammatory reaction in the infected ear. Representative photomicrographs of ear histological sections from C57BL/6 mice submitted to HSB and AIN93-G diets and infected with *L*. *major*. (A, B and C) photomicrography from control mice 2, 4 and 8 weeks post infection. The cellular infiltration is predominantly polymorphonuclear (Insert in A, B), focal and discrete to moderate infiltration in the papillary dermis and deep dermis accompanied by mild to moderate thickening of the dermis; erosion and ulceration of the epidermis, predominantly polymorphonuclear inflammatory process with discrete mast cell hyperplasia (Insert in C) and thickening of the dermis, moderate to severe at eight weeks post infection. (D, E and F) photomicrography from HSB-fed mice 2, 4 and 8 weeks post infection. Ulcerated epidermis, predominantly polymorphonuclear and focal inflammatory process in the dermis and hypodermis, with thickening of the dermis, both of intense character, and tissue parasitism (Insert in D) in animals belonging to the obese group, 2 weeks post infection; moderate thickening of the dermis and inflammatory infiltrate also moderate, predominantly polymorphonuclear (Insert in E) and focal, in the papillary dermis, deep dermis and hypodermis 4 weeks post infection; ulcerated epidermis, predominantly polymorphonuclear and focal inflammatory process in the dermis and hypodermis with thickening of the dermis, both moderately characterized with moderate mast cell hyperplasia (Insert in F), 8 weeks after infection. Hematoxylin-Eosin. Bar in the normal image = 25mm.

In order to detect the effects of obesity on the course of infection with *L*. *major*, C57BL/6 mice were infected in the left ear with metacyclic promastigotes, 4 weeks after the HSB-diet consumption. Ear thicknesses in obese mice were significantly higher than in control mice from the fifth-week post infection (Fig 1C). Parasite burden was also checked 2, 4 and 8 weeks post-infection. Two weeks post-infection and eight weeks post infection obese mice presented higher parasitism (Fig 1D). At 8 weeks after infection, obese mice presented a more ulcerative lesion with extensive necrosis and tissue damage when compared to the control group (Fig 1E). Histological analysis of ear samples showed differences in the inflammatory profile of the lesions between obese and control mice. Control mice showed less inflammatory infiltration at all times post-infection analyzed (Fig 2A, 2B and 2C) comparing to obese mice at the same time points (Fig 2D, 2E and 2F). It is also important to note that there are visible parasites only in the tissue from obese mice (inserts—Fig 2D and 2E). At 8^th^-week post-infection, obese mice presented greater cellular infiltrate, including polymorphonuclear cells, and mast cell hyperplasia (Fig 2F).

### Obesity induced increased production of *L*. *major* specific IgG1

**Fig 3:**
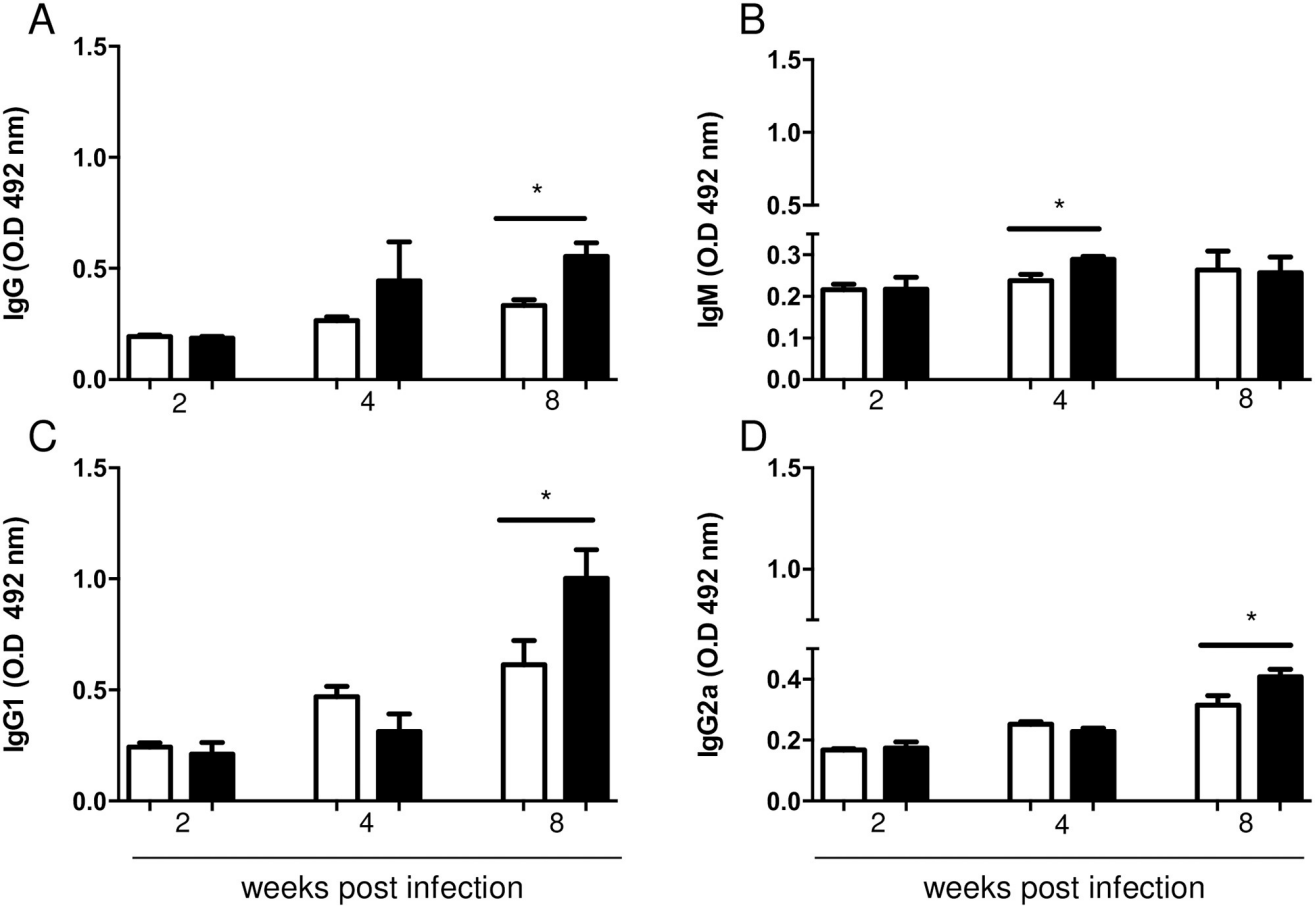
Diet-induced obesity increases the concentration of *L*. *major* specific immunoglobulins in C57BL/6 serum. Total IgG (A), IgM (B), IgG1 (C) and IgG2a (D) were measured in sera from mice fed control (AIN93G) and hypercaloric (HSB) diet. ELISA was performed at 2, 4 and 8 weeks of infection. For sensitization, *L*. *major* antigen at 20 μg/mL was used. To measure IgG, sera were diluted 1:1000, and 1:100 for IgG1, IgG2a and IgM. Data are represented as average ± SD. Statistical analysis was performed by Student´s *t* test (*p<0.*05*; **p<0.*005*). Results are representative of at least two independently experiments, n = 4 mice/group.

In the 8^th^ week of infection, obese mice presented higher levels of specific circulating IgG, IgG1 and IgG2a, when compared to the levels found in mice from the control group (Fig 3A, 3C and 3D). Serum IgM levels were higher in obese mice only on the 4^th^ week. There was an increase in total IgG, IgG1 and IgG2a levels on the 8^th^ week of infection in obese mice when compared to their control counterparts suggesting that increased antibody production during obesity may be associated with a lower efficiency to kill *Leishmania*.

### Obese C57BL/6 mice showed no impairment in IFN-gamma production but had increased IL-17 production by auricular draining lymph node cells

**Fig 4:**
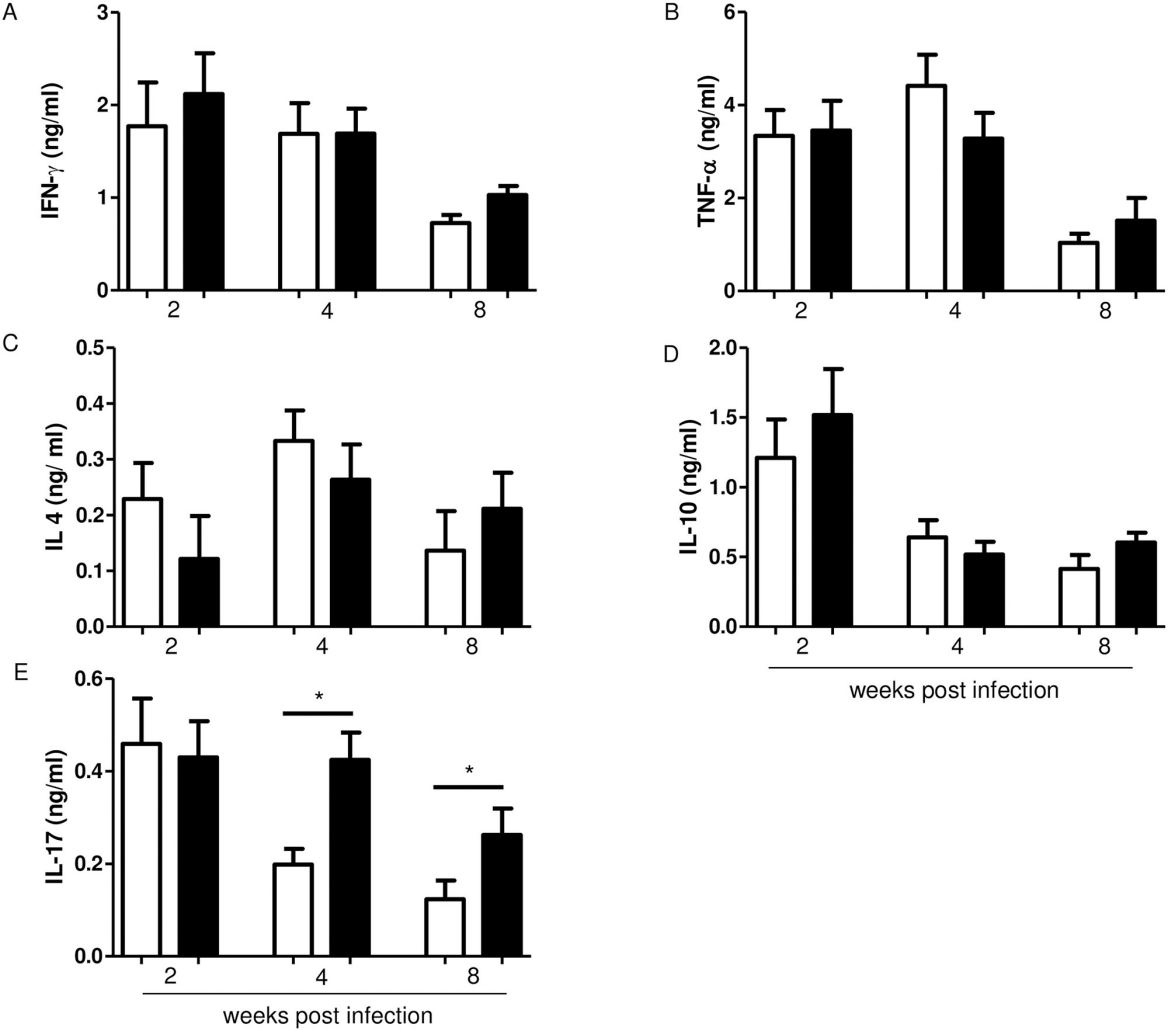
Cytokine production by lymph node cells in culture. Cytokine concentrations were measured by ELISA in cell culture supernatants stimulated *in vitro* with 50 μg/mL of *L*. *major* antigen. Cells were collected 2, 4 and 8 weeks after infection and adjusted for 5x10^6^/mL of culture and incubated during 72h. (A) IFN-gamma; (B) TNF-alfa; (C) IL-4; (D) IL-10 and (E) IL-17. Data are represented as average ± SD. Statistical analysis was performed by Student´s *t* test (* p<0.05). Results are representative of at least two independent experiments, n = 4 mice/group.

Both control and obese mice produced equivalent levels of IFN-gamma by cells from auricular draining lymph node (Fig 4A). The same result was observed for TNF-alfa (Fig 4B). These data suggest that the increase in parasite burden observed in obese mice was not related to a deficient Th1 response.

We also evaluated the production of IL-4, IL-10, and IL-17 in the supernatant of cultured cells from auricular draining lymph nodes of the infected ear. There was no difference in IL-4 or IL-10 production between obese mice and control mice (Fig 4C and 4D). Interestingly, IL-17 production was elevated on the second week post-infection in both groups. However, in obese mice, IL-17 levels stayed higher up to the 8^th^ week of infection in obese mice, whereas it decreased in the control mice by the same time. Auricular lymph nodes cells from non-infected mice were stimulated *in vitro* with ConA, and we observed significantly increased concentrations only for IL-10 in the supernatants (S2 Fig).

Cytokine secretion in adipose tissue was also evaluated and no difference was found for IFN-gamma or IL-17. As expected, TNF-alfa levels were increased in adipose tissue from obese mice before infection. Two weeks after infection, TNF-alfa levels decreased, and there was no difference between control and obese mice at 4^th^ and 8^th^ weeks post infection. IL-10 production in adipose tissue also presented some differences between obese and control mice. In obese mice, IL-10 levels were higher before infection and four weeks after infection (S3 Fig). IL-17 production is higher in obese mice at different organs, even without infection (S4 Fig).

### Macrophages from obese mice had higher arginase activity and higher parasitism when infected *in vitro* with *L*. *major*

**Fig 5:**
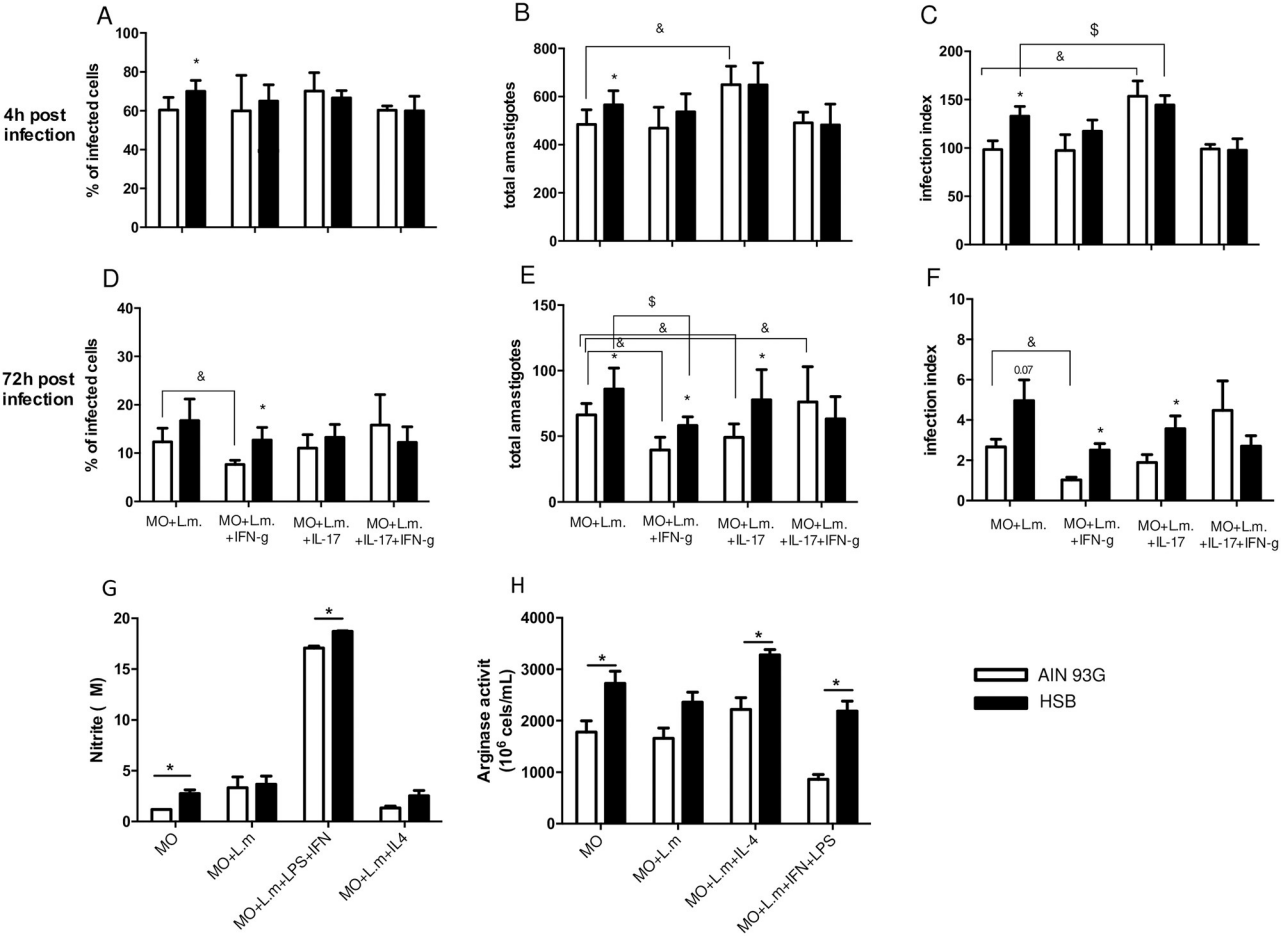
*In vitro* macrophage infection. To measure the percentage of infected macrophage (MO) and the amastigote number in infected MO, cells were collected from the peritoneal cavity after thioglycotate stimulation and cultured at 1x10^5^/ml. The infection was performed with 5 total non opsonized promastigotes of *L*. *major* per MO 24h after adhesion. Parasite counts were performed at 4 and 72 hours post infection. Arginase and NO were measured 72h after infection, as described in Materials and Methods. (A and D) Percentage of infected MO; (B and E) Amastigote number per infected MO and (C and F) Infection index [(MO*100/infected MO)*(amastigotes/infected MO)]; (G) NO production; and (H) Arginase activity. Data are represented as average ± SD. Statistical analysis was performed by Student´s *t* test (*) means p<0.05 between MO from control mice versus obese mice; (^&^) means p<0.05 comparing MO from control group without stimulus and with different culture conditions and (^$^) means p<0.05 comparing MO from obese group without stimulus and with different culture conditions. n = 6.

To evaluate the leishmanicidal capacity of macrophage from obese mice, we analyzed the degree of infection of macrophages at 4 and 72 hours post *in vitro* infection with *L*. *major*. At 4 hours post infection, macrophages from obese mice had a higher percentage of infected macrophages as well as a higher number of amastigotes per macrophage, and also a higher infection index when compared to macrophages from control mice (Fig 5A, 5B and 5C). Of note, *in vitro* infection was performed using non opsonized total promastigotes, and that could explain why we found fewer amastigotes 72h after infection than 4h post-infection [31]. At 72 hours post infection, macrophages from obese mice harbor more amastigotes than macrophages from control mice (Fig 5E), in both experimental situations, stimulated with IFN-gamma or with IL-17. To verify whether IL-17 would impair the killing of amastigotes *in vitro*, we stimulated the cultures with IL-17. Macrophages from control mice stimulated with IFN-gamma had a decrease in number of amastigotes, percentage of infected cells and also infection index, 72h post infection. In the obese macrophages, the IFN-gamma stimulus decreased the number of amastigotes, but not the percentage of infected cells or the infection index, and in the IL-17 stimulated wells macrophages from obese mice are similar with the no stimulated macrophages. Macrophages from control mice, when cultured *in vitro* with IL-17, had similar infection index and percentage of infected cells compered with no stimulated macrophages 72h post-infection, differently from when they were stimulated with IFN-gamma. These data suggest that IL-17 may interfere with parasite elimination. On the other hand, addition of IFN-gamma in the culture of macrophages from obese mice also decreased the number of amastigotes and the infection index, but not the percentage of infected cells.

We also investigated the production of NO and arginase activity by macrophages from obese mice. Macrophages from obese mice without any stimulus or stimulated with IFN-gamma and LPS produced slightly more NO than cells from the control group (Fig 5G). However, arginase activity was also higher in non-stimulated cells from the obese mice, as well as in cells infected with *L*. *major*. IL-4 stimulation did not increase arginase activity, but arginase activity remained higher in obese mice. Macrophages from obese mice also displayed higher arginase activity upon activation with IFN-gamma and LPS (Fig 5H). Therefore, our data suggest that obesity increases arginase activity in macrophages.

### The absence of IL-17 receptor abolished the effect of obesity in the profile of *L*. *major* infection

**Fig 6:**
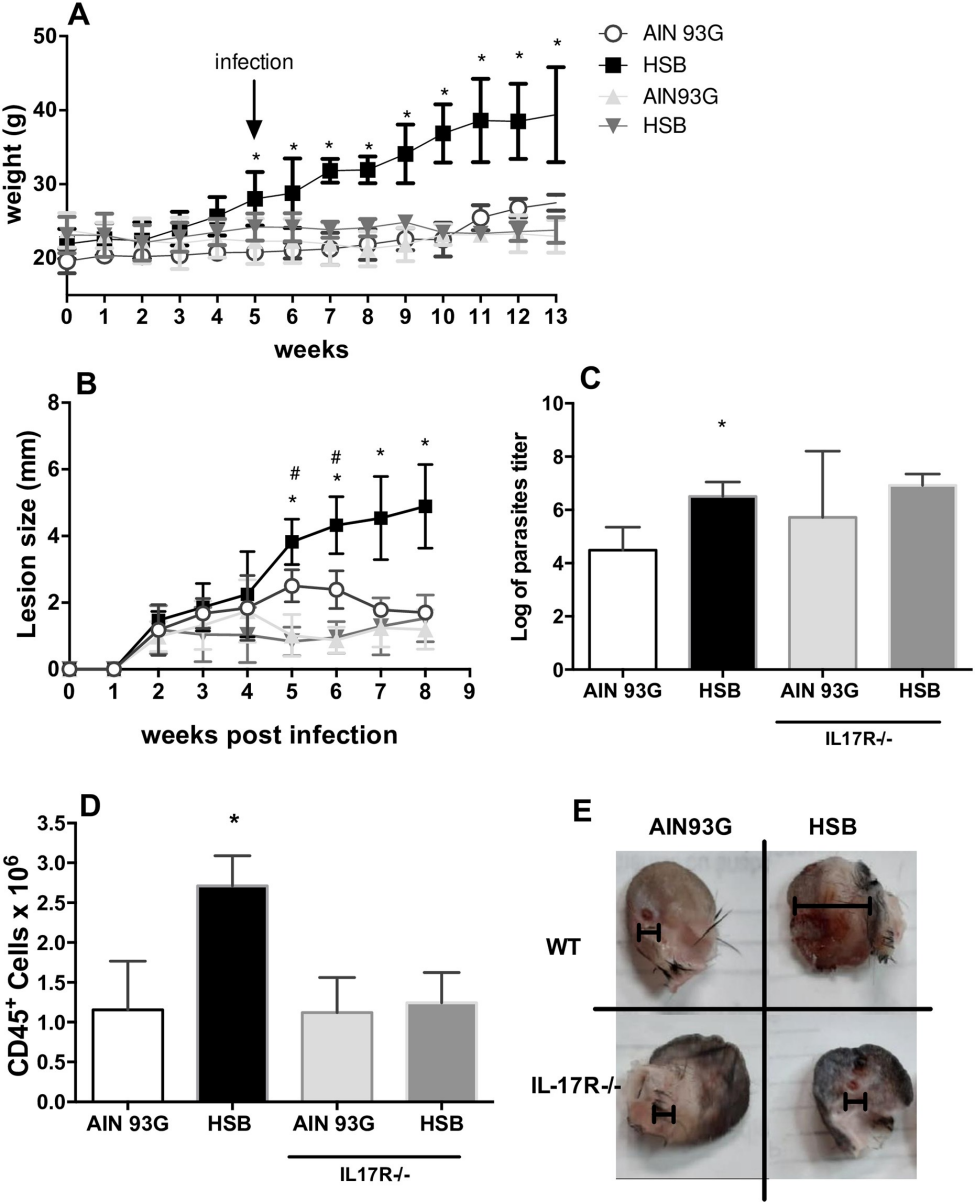
HBS fed IL-17R-/- mice do not show difference in *L*. *major* infection profile compared with control AIN93G fed mice. Wild type and IL17R-/- C57BL/6 mice were fed either control (AIN93G) or hypercaloric (HSB) diets *ad libitum* for four weeks. On the fifth week mice were infected with 1x10^6^ metacyclic *L*. *major* in the ear. (A) Weekly measurement of body weight. (B) Weekly measurement of infected lesion during eight weeks of infection. (C) Parasite titer in the infected ear, measured by limiting dilution. (D) Absolute number of CD45+ cells in the draining lymph node. (E) Representative pictures of ears lesions of IL17R-/- and WT at week 8 post infection. Data are represented as average ± SD. Statistical analysis was performed by Anova test (*p<0.05; **p<0.005). n = 4/ group.

To further characterize the role of IL-17, we fed IL-17R-/- mice with HSB and AIN93G diet and infected them with *L*. *major*. The lack of signaling through the IL-17 receptor changed the effect of obesity in *L*. *major* infection. Wild type (WT) but not IL17R-/- mice fed HSB diet gained weight when compared to IL-17R-/- and WT mice fed control diet (Fig 6A). Furthermore, HBS fed and AIN93G fed IL-17R-/- mice presented a similar course of infection shown by their lesion development (Fig 6B and 6E). Interestingly, at the 8^th^ week of infection IL17R-/- mice had similar parasite burdens regardless of the diet they were fed (Fig 6C). In concert with the similarity in lesion size (Fig 6B), IL-17R-/- mice did not show increased accumulation of CD45+ cells (leukocytes) in auricular draining lymph nodes (Fig 6D), suggesting that IL-17 signaling was needed for the increase in lymph node leukocyte numbers and for lesion formation during obesity. Both HBS fed and AIN93G fed IL-17R-/- mice showed non-ulcerative and non-necrotic lesions at the 8^th^ week post infection (Fig 6E).

## Discussion

The impact of obesity in the immune response to cutaneous leishmaniasis has never been described. Our results showed that C57BL/6 mice with diet-induced obesity present larger lesions than control lean mice, and these lesions contained more parasites. We observed no difference in IFN-gamma, IL-4 and TNF-alfa production by cells from the auricular draining lymph nodes of these mice, but there was an increase in the levels of IL-17. Moreover, peritoneal macrophages from obese mice were less efficient in killing amastigotes than macrophages from control mice, and addition of IL-17 to macrophage cultures decreased their ability to control infection compared with ones stimulated with IFN-gamma. To further clarify the role of IL-17 in this process, we fed IL-17 receptor-deficient C57BL/6 mice with either hypercaloric HBS or control AIN93G diet, and infected them with *L*. *major*. Interestingly, obese IL-17R-/- did not have augmented lesions, but presented high parasite load, and no difference in infection was observed between HBS fed or AIN93G fed mice.

There is a gap in understanding how obesity affects the course of intracellular infections, including parasite infections. In accordance with the negative association between obesity and infectious diseases already described [22,32,33], our study showed that obese mice had higher parasite burden than lean mice, failing to control parasite growth. In addition, lesions in obese mice were larger and more ulcerative than the lesion in the control group. Adipose tissue produces cytokines, adipokines, and chemokines which alter the immune response, affecting the recruitment of inflammatory cells, such as macrophages, neutrophils and dendritic cells [34]. It is known that obesity also affects T cell differentiation and function, increasing pro-inflammatory cytokine production [35,36]. Considering that obesity is associated with a “low-grade” inflammation, one reasonable hypothesis could be that in the specific case of cutaneous leishmaniasis, where macrophages require an inflammatory environment to control parasites, obesity would improve the immune response against *L*. *major*. However, our data do not support this hypothesis. The histological data also detected a higher parasite burden in obese C57BL/6 mice when compared to mice from the control group further indicating that obesity interferes with control of parasite growth. Interestingly, lesions in obese mice showed increased cellular infiltration; however, the inflammatory cells had poor ability to eliminate the parasites.

We also measured the antibody response to *L*. *major* in sera and found higher levels of IgG and IgG1 in obese C57BL/6 mice than control mice after eight weeks of infection. As described in previous studies, susceptibility to *L*. *major* infection is associated with isotype switching to IgG1 [37,38]. Antibody levels are directly related to parasite number, as antibodies may form immune complexes that bind to Fcγ receptors (FcγR), inhibiting the proinflammatory activity of macrophages, without impairing phagocytosis [39]. Interestingly, despite favoring phagocytosis, IgG fails to protect against *L*. *major*, and even worse, contributes to the pathogenicity itself [40]. Indeed, it has been reported that phagocytosis of IgG-opsonized amastigote forms induces the activation of signaling pathways leading to the production of IL-10 by macrophages [38].

To understand why obese C57BL/6 mice had more severe lesions than control mice, we also evaluated cytokine production. Obesity did not affect the production of IL-4 in C57BL/6 mice. This result differs from previous data that associate obesity with susceptibility to asthma and allergies, driven by higher IL-4 secretion and Th2 differentiation [41,42]. We also showed no difference in TNF-alfa and IFN-gamma levels at the time points measured. Obese mice did not show overproduction of proinflammatory cytokines by cells from the draining lymph nodes. In response to parasite antigens, it was expected a higher production of pro-inflammatory cytokines by obese mice, as it was observed for infection with *Plasmodiun berguei* [19] and also for infection with *Leishmania chagasi* [22].

The role of IL-17 in leishmaniasis has been a subject of debate. We found a higher IL-17 production by lymph node cells of obese mice, which presented a more severe lesion with higher parasite burden than the control group. Classically, IL-17 production is associated with neutrophil recruitment and often is related to pro-inflammatory response in various diseases, including autoimmune disorders [43], fungal [44] and bacterial infections [45]. However, recent studies have questioned whether IL-17 function is restricted to pro-inflammatory action [46]. IL-17 is produced mainly by Th17 cells, which could be stimulated by a different combination of cytokines, including TGF-β, IL-6, IL-23 and IL-1β [43]. Obesity alters the cytokine profile in adipose tissue and in serum, and IL-17 seems to have a significant role in obesity. Previous studies have shown that production of this cytokine is elevated in obesity, both in humans and mice [47,48]. In line with these reports, our results showed that cells from draining lymph nodes from infected obese mice secrete higher levels of IL-17. Studies on cutaneous leishmaniasis have correlated IL-17 release with increased pathogenicity in cutaneous leishmaniasis in mice [49,50]. Results obtained using mice IL-17R-/- showed that these mice had less severe lesions then wild type mice with lower numbers of leukocytes in the draining lymph nodes; however the parasite burden was as high as in obese WT mice, with no difference between HBS fed or AIN93 fed mice. Therefore, IL-17 seems to play a critical role in lesion and ulcer formation in obese mice during *L*. *major* infection.

We also performed *in vitro* infection of macrophages with *L*. *major* to assess the efficiency of macrophages from obese C57BL/6 mice to kill the parasites. There was an increased frequency of infected cells among macrophages from obese mice 4 hours post infection, and a larger number of amastigotes/cell in macrophages from obese mice 4 and 72 hours post infection. These results are in line with higher arginase activity detected in macrophages from obese mice. Interestingly, Sousa and coworkers found that IL-17 increases arginase activity, and favor parasite growth in BALB/c mice infected with *L*. *amazonensis* [51]. Arginase activity is increased in mice susceptible to cutaneous leishmaniasis, and this enzyme utilizes arginine as a substrate to induce polyamine production instead of NO [52]. Interestingly, studies on inflammatory bowel disease have demonstrated that IL-17 induces an alternative activated macrophages response. Moreover, IL-17KO C57BL/6 mice expressed lower levels of mRNA coding for molecules associated with alternative activated macrophages activity, including arginase 1 [53]. Another study showed that IL-17 induces arginase 1 production in a model of human Papillomavirus [54]. In this case, arginase 1 would be active in the macrophages in our experiments. Our *in vitro* results showed an environment where IFN-gamma would still be inducing the parasite control in obese macrophages. However, IL-17 production decrease, even a little, the leishmanicidal activity of macrophages from obese mice and could further facilitated parasite growth by increasing arginase activity.

To test the hypothesis that IL-17 would impair leishmania killing by macrophages, we infected macrophages stimulated *in vitro* with IL-17. IL-17 interfered with parasite elimination by macrophages from control and obese mice. Moreover, addition of this cytokine decreased parasite killing by control macrophages activated with IFN-gamma.

To further confirm our hypothesis, we performed an experiment with IL-17 receptor deficient mice. These mice did not show weight gain when fed HSB diet and no difference was observed between lean and obese mice infected with *L*. *major*. It was reported before that IL-17R-/- mice did not gain weight when fed a high fat diet because they developed an inflammatory reaction in the small intestine that allowed bacterial translocation [55].

It was already observed that IL-17-deficient BALB/c mice developed smaller lesions despite only a modest reduction in parasite loads [49] when infected with *L*. *major*, the same profile we observed in obese IL-17R-/- mice that controlled lesion sizes but not parasite burdens. It is not in the literature that the wound induced by *Leishmania ssp*. is not always related with parasite burden [56,57].

In the present work, we propose IL-17 as an alternative cytokine that may determine the fate of the lesion formation in response to *L*. *major* in mice that received hypercaloric diet. We showed that diet-induced obesity, a condition associated with IL-17 production, increased the susceptibility of C57BL/6 mice, a mouse strain genetically resistant to *L*. *major* infection. Therefore, IL-17 could be a potential candidate to explain diverse comorbidities associated with obesity and its role in models of infection and ulcerated wound.

Taken together, our results showed that diet-induced obesity in C57BL/6 mice decreased their capacity to control infection by *L*. *major*. This might be related to the induction of IgG1 secretion, IL-17 production and impaired capacity of macrophages to control parasite growth. The present results provide novel clues about the relationship between obesity and leishmanial infection in a time when infection is now being added to the list of health risks associated with obesity.

## Supporting information

S1 Fig Metabolic evaluation in mice fed HSB diet before and after *L*. *major* infection. (A) Fasting glycaemia. (B) Glucose oral tolerance test performed eight weeks post infection. Mice were fasted for six hours and received a 30% glucose solution. Blood was taken at time 0 and 15, 30, 60 and 90 minutes after administration of the glucose solution. (C) Total blood cholesterol eight weeks post infection. (D) Serum leptin concentration measured by ELISA (8 weeks post infection and 12 weeks post diet consumption). Statistical analysis was performed by Student´s *t* test (* p<0.05; ** p<0.005 and *** p<0.0005). Data are represented as average ± SD. Results are representative of at least 4 independently experiments, n = 4mice/group.
(TIF)

S2 Fig Cytokine production by auricular lymph node cells in culture from non-infected mice. Cytokine concentrations were measured by ELISA in cell culture supernatants stimulated *in vitro* with 10μg/ml of ConA. Cells were collected and adjusted for 5x10^6^/mL of culture and incubated during 72h. Data are represented as average ± SD. (A) IFN-γ; (B) IL-4; (C) IL-10 and (D) IL-17. n = 5 mice/group.
(TIF)

S3 Fig Cytokine profile in the adipose tissue extract from C57BL/6 mice infected with *L*. *major*. The peritoneal adipose tissue extracts were prepared (100 mg/mL of buffer) and ELISA was performed to measure concentrations of IFN-gamma, TNF-alfa, IL-10, IL-17 and IL-4. (A) IFN-gamma; (B) TNF-alfa; (C) IL-10; (D) IL-17. IL-4 values were below the detection limit. Data are represented as average ± SD. Statistical analysis was performed by Student´s *t* test (*p<0,05). Results are representative of at least two independently experiments, n = 4 mice/group.
(TIF)

S4 Fig IL-17 concentration in different organs without infection. Spleen cells were collected and stimulated *in vitro* with 10μg/mL of ConA 4 (A) and 12 (B) weeks after mice consume AIN93G or HSB diet. The peritoneal adipose tissue extracts (100mg/ml of buffer) were prepared 12 weeks after mice consume AIN93G or HSB diet (C) and serum were collected also 12 weeks after mice consume AIN93G or HSB diet (D). ELISA was performed to measure concentrations of IL-17. Data are represented as average ± SD. n = 4 or 5 mice/group.
(TIF)
