# Supplementary figures and images for: Obesity impairs resistance to Leishmania major infection in C57BL/6 mice
Source: PLoS Negl Trop Dis. 2020 Jan 10;14(1):e0006596. doi: 10.1371/journal.pntd.0006596 (PMC6953764; doi:10.1371/journal.pntd.0006596)

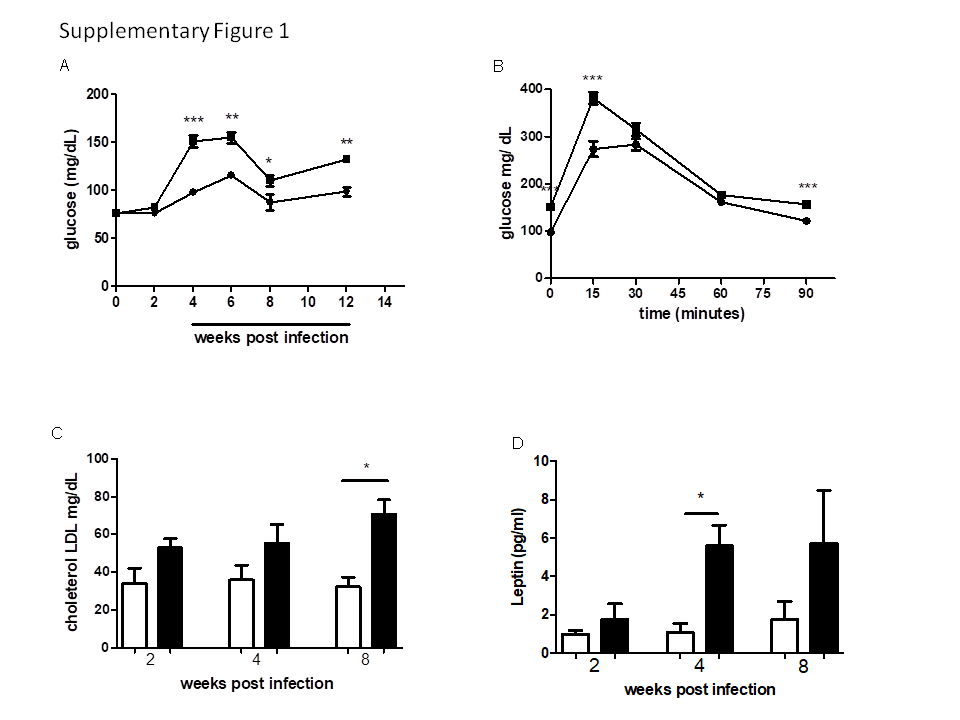

Supplement: S1 Fig — (A) Fasting glycaemia. (B) Glucose oral tolerance test performed eight weeks post infection. Mice were fasted for six hours and received a 30% glucose solution. Blood was taken at time 0 and 15, 30, 60 and 90 minutes after administration of the glucose solution. (C) Total blood cholesterol eight weeks post infection. (D) Serum leptin concentration measured by ELISA (8 weeks post infection and 12 weeks post diet consumption). Statistical analysis was performed by Student´s t test (* p<0.05; ** p<0.005 and *** p<0.0005). Data are represented as average ± SD. Results are representative of at least 4 independently experiments, n = 4mice/group. (TIF) [file pntd.0006596.s001.tif]

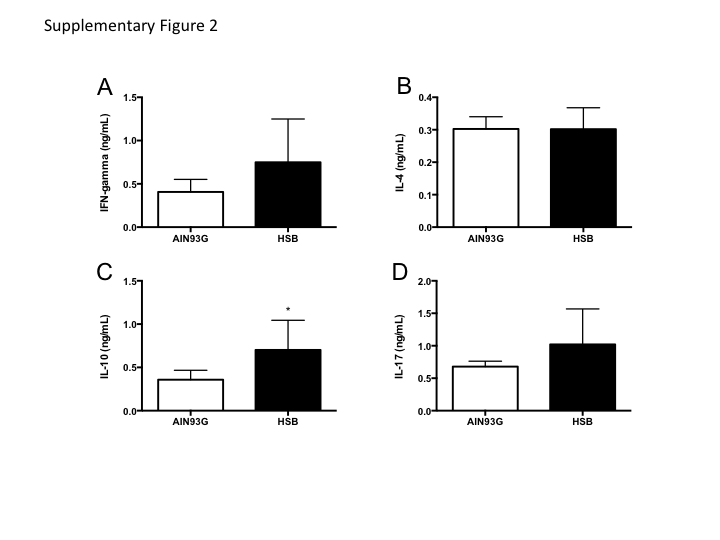

Supplement: S2 Fig — Cytokine concentrations were measured by ELISA in cell culture supernatants stimulated in vitro with 10μg/ml of ConA. Cells were collected and adjusted for 5x10^6^/mL of culture and incubated during 72h. Data are represented as average ± SD. (A) IFN-γ; (B) IL-4; (C) IL-10 and (D) IL-17. n = 5 mice/group. (TIF) [file pntd.0006596.s002.tif]

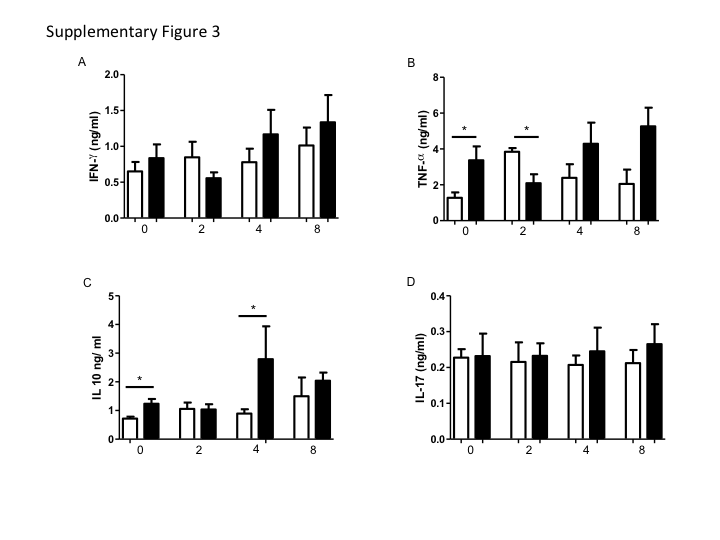

Supplement: S3 Fig — The peritoneal adipose tissue extracts were prepared (100 mg/mL of buffer) and ELISA was performed to measure concentrations of IFN-gamma, TNF-alfa, IL-10, IL-17 and IL-4. (A) IFN-gamma; (B) TNF-alfa; (C) IL-10; (D) IL-17. IL-4 values were below the detection limit. Data are represented as average ± SD. Statistical analysis was performed by Student´s t test (*p<0,05). Results are representative of at least two independently experiments, n = 4 mice/group. (TIF) [file pntd.0006596.s003.tif]

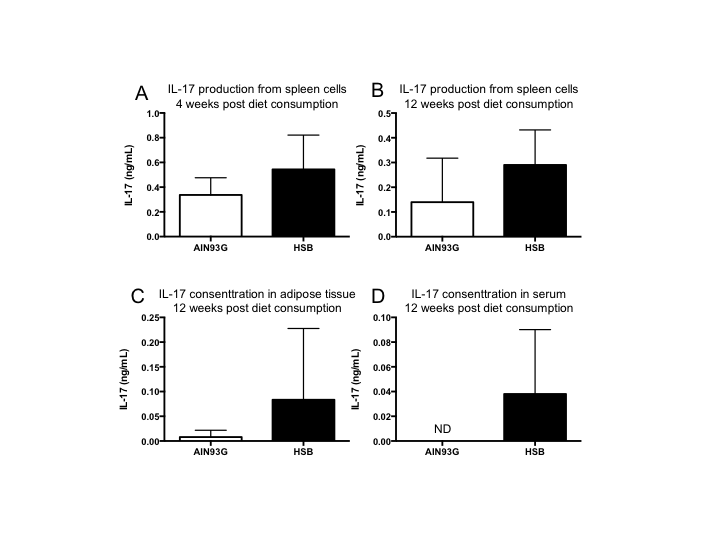

Supplement: S4 Fig — Spleen cells were collected and stimulated in vitro with 10μg/mL of ConA 4 (A) and 12 (B) weeks after mice consume AIN93G or HSB diet. The peritoneal adipose tissue extracts (100mg/ml of buffer) were prepared 12 weeks after mice consume AIN93G or HSB diet (C) and serum were collected also 12 weeks after mice consume AIN93G or HSB diet (D). ELISA was performed to measure concentrations of IL-17. Data are represented as average ± SD. n = 4 or 5 mice/group. (TIF) [file pntd.0006596.s004.tif]
